# Morbidity and mortality of COVID-19 negatively associated with the frequency of consanguineous marriages, an ecologic study

**DOI:** 10.1186/s43042-022-00218-8

**Published:** 2022-01-21

**Authors:** Mostafa Saadat

**Affiliations:** Department of Biology, College of Sciences, Shiraz University, 71467-13565 Shirazm, Iran

**Keywords:** Consanguineous marriage, COVID-19, Ecologic study

## Abstract

**Background:**

Union between second cousins and closer relatives is called consanguineous marriage. Consanguineous marriage is associated with increased risk of autosomal recessive diseases and several multifactorial traits. In order to evaluate the association between prevalence/mortality of COVID-19 and the frequency of consanguineous marriage, the present ecologic study was carried out. For the present study, data of prevalence (per 10^6^ people) and mortality (per 10^6^ people) and number of performed laboratory diagnostic test (per 10^6^ people) of COVID-19 disease at four time points (December 2020; March, August and October 2021) of 65 countries were used.

**Results:**

Univariable correlation and generalized estimating equation analysis were used. In analysis, prevalence and mortality of COVID-19 were used as dependent variables and human development index, number of performed diagnosis test and the mean of inbreeding coefficient (α-value) were introduced into model as covariates, and time point was used as a factor in analysis. The square root (SR) of prevalence (*P* = 0.008) and SR-mortality (*P* < 0.001) of COVID-19 negatively associated with the log-transformed of α-value.

**Conclusions:**

The present finding means that in countries with high levels of consanguineous marriages, the prevalence of COVID-19 and mortality due to COVID-19 were lower than countries having low level of marriage with relatives.

## Background

In genetics, union between second cousins and closer relatives is called consanguineous marriage [1]. Previously it has been shown that consanguineous marriage is a long-standing social custom which depends with numerous factors, such as religious, socio-economic and demographic variables [2–4]. The frequency of consanguineous marriage has geographical distribution; it has high prevalence in several Asia and Africa countries. Iranian populations showed high levels of consanguineous marriages [5–7].

Numerous studies have shown that this type of marriage is associated with increased risk of autosomal recessive diseases and several multifactorial traits [1, 8–15]. It is well established that primary immunodeficiency diseases are genetically heterogeneous group and are associated with parental consanguinity [16]. There is significant association between parental consanguineous marriage and the risk of some infections [17, 18]. There is about one-fifth of the world’s population living in countries where marriage with biological relatives is prevalent. Therefore, for countries with high prevalence of consanguineous marriage, the association between frequency of consanguineous marriage and the Coronavirus Disease 2019 (COVID-19) is highly important. To the best of our knowledge, there is no study regarding the above-mentioned association; therefore, the present ecologic study was carried out.

## Methods

Prevalence (per 10^6^ people) and mortality (per 10^6^ people) and number of performed laboratory diagnostic test (per 10^6^ people) of COVID-19 disease at four time points (December 31, 2020; March 19, 2021; August 31, 2021 and October 25, 2021) were obtained from the Web site www.worldometers.info/coronavirus (Table 1).

**Table 1:** Prevalence and mortality of COVID-19 (at four time points), α-value and human development index (HDI) in 65 countries used in the study

Country	α (× 10^–4^)	HDI	Prevalence (per 10^6^ people)				Mortality (per 10^6^ people)				Number of diagnostic tests performed (per 10^6^ people)			
			I	II	III	IV	I	II	III	IV	I	II	III	IV
Afghanistan	277	0.511	1334	1418	3837	3893	56	62	178	181	5090	8335	18,818	19,232
Argentina	3	0.845	35,801	49,128	113,525	115,468	952	1197	2448	2533	105,970	181,382	484,192	543,219
Australia	1	0.944	1107	1135	2084	6189	35	35	39	64	438,894	587,753	1,220,092	1,635,789
Bahrain	165	0.852	53,547	77,161	153,973	155,486	203	283	784	783	1,367,218	1,941,619	3,352,349	3,837,157
Bangladesh	45	0.632	3103	3418	9007	9398	46	52	157	167	19,500	26,336	53,592	61,392
Belgium	3	0.931	55,466	70,774	101,543	113,449	1674	1946	2178	2220	594,130	888,169	1,600,817	1,814,678
Bolivia	3	0.718	13,475	22,309	41,361	42,915	778	1021	1554	1591	35,029	70,567	192,444	211,825
Brazil	21	0.765	35,983	55,594	96,949	101,310	914	1360	2709	2824	134,070	133,871	265,480	297,262
Canada	4	0.929	15,337	24,410	39,320	44,597	412	596	706	755	363,376	694,395	1,063,272	1,198,495
Chile	6	0.851	31,719	47,733	84,874	87,082	865	1148	1913	1949	336,033	541,582	1,050,216	1,200,325
China	27	0.761	60	63	66	67	3	3	3	3	111,163	111,163	111,163	111,163
Colombia	35	0.767	32,113	45,337	95,296	96,764	845	1205	2425	2463	159,013	237,702	469,190	514,898
Costa Rica	11	0.810	33,087	41,328	90,075	108,078	427	565	1069	1355	96,770	147,838	389,729	489,646
Croatia	1	0.851	51,519	62,543	91,762	109,531	958	1405	2045	2225	249,025	357,598	624,180	745,949
Cuba	5	0.783	1048	5754	57,680	83,672	13	34	469	724	130,412	242,869	701,100	917,622
Czech Republic	1	0.900	67,054	135,193	156,464	161,273	1087	2269	2833	2853	352,869	980,182	3,346,877	3,720,067
Ecuador	13	0.759	11,954	17,363	27,915	28,656	789	920	1796	1831	42,113	61,332	98,334	106,401
Egypt	123	0.707	1337	1872	2758	3104	74	111	160	175	9681	9643	29,344	35,220
El Salvador	14	0.673	7068	9606	14,681	17,198	205	303	447	548	95,594	123,973	184,668	206,332
France	2	0.901	40,100	63,962	103,385	108,872	989	1402	1749	1795	535,994	894,795	1,906,567	2,309,763
Guinea	131	0.477	1031	1387	2178	2253	6	8	25	28	7995	12,430	39,642	41,545
Honduras	11	0.634	12,203	18,050	33,581	37,026	314	439	882	1009	30,696	48,518	96,817	106,821
Hungary	1	0.854	33,428	57,021	84,339	88,337	989	1850	3121	3175	275,409	435,797	682,588	757,183
India	238	0.645	7417	8315	23,507	24,468	107	115	315	326	124,060	166,492	373,653	429,788
Indonesia	95	0.718	2703	5262	14,771	15,291	81	143	480	516	26,748	43,419	116,350	163,285
Iran	185	0.783	14,493	21,075	58,563	68,712	653	727	1265	1468	89,515	142,043	330,974	381,937
Iraq	225	0.674	14,636	19,204	45,751	49,457	315	341	505	555	111,811	185,560	347,760	379,122
Ireland	1	0.955	18,483	46,072	70,462	86,168	451	919	1018	1072	478,239	763,477	1,282,683	1,576,220
Italy	4	0.892	34,877	55,174	75,217	78,610	1227	1726	2141	2185	440,252	765,907	1,392,283	1,678,211
Japan	13	0.919	1824	3587	11,652	13,630	27	69	127	144	38,422	72,856	172,729	207,359
Jordan	200	0.729	28,720	50,749	77,234	82,527	374	555	1009	1061	309,687	525,879	896,743	1,042,639
Kuwait	205	0.806	35,002	50,188	94,351	94,765	217	280	557	565	291,671	452,251	871,679	1,085,320
Lebanon	91	0.744	26,653	63,838	88,701	94,036	216	833	1186	1247	292,040	495,158	700,912	704,511
Libya	209	0.724	14,495	21,670	44,266	50,646	214	358	608	719	78,997	117,011	218,213	252,516
Malaysia	47	0.810	3469	10,105	53,161	74,034	14	38	507	866	102,668	216,935	691,764	1,019,218
Mexico	1	0.779	10,909	16,799	25,602	28,944	964	1514	1981	2191	27,751	44,955	74,504	85,806
Mongolia	63	0.737	368	1405	64,025	104,733	0	2	281	498	181,857	634,290	1,092,266	1,203,868
Morocco	89	0.686	11,828	13,192	23,008	25,193	199	235	338	390	120,046	157,123	239,820	267,224
Nepal	203	0.602	8864	9345	25,639	27,150	63	102	361	381	65,733	75,594	131,621	146,752
Netherlands	1	0.944	46,460	69,164	112,990	121,495	666	947	1048	1066	336,388	406,153	965,933	1,046,785
Nigeria	242	0.539	420	770	907	992	6	10	12	14	4543	8030	13,108	15,500
Norway	2	0.957	9107	15,691	29,279	36,774	80	119	149	163	512,352	783,127	1,312,037	1,475,055
Oman	169	0.813	24,920	28,688	57,501	57,645	290	312	773	779	110,512	298,161	294,829	4,737,668
Pakistan	282	0.557	2151	2765	5136	5603	45	61	114	125	30,023	43,276	78,612	90,633
Panama	6	0.815	56,749	80,269	104,123	107,102	925	1383	1607	1660	300,323	471,752	834,635	915,784
Peru	16	0.777	30,574	43,595	64,168	65,414	1135	1498	5918	5959	166,330	258,036	499,973	560,933
Philippines	3	0.718	4297	5859	17,881	24,766	84	117	301	376	61,243	86,269	168,302	203,555
Portugal	9	0.864	40,630	80,255	102,138	106,861	678	1647	1746	1786	548,976	857,034	1,672,408	1,934,376
Qatar	275	0.848	51,226	61,506	82,892	84,992	87	97	214	217	442,127	589,354	892,029	996,997
Saudi Arabia	223	0.854	10,339	10,917	15,361	15,434	177	187	241	247	313,818	413,298	774,341	847,162
Singapore	20	0.938	9977	10,227	11,453	29,745	5	5	9	56	923,868	1,369,279	3,010,388	3,496,485
Slovakia	1	0.860	32,877	63,379	72,294	83,731	392	1628	2297	2364	261,676	410,607	607,648	706,312
Slovenia	3	0.917	58,775	98,349	128,506	154,195	1297	1905	2140	2257	324,385	480,333	695,973	813,725
South Africa	16	0.709	17,713	25,658	46,157	48,428	477	870	1367	1475	110,737	160,173	273,773	304,837
Spain	6	0.904	41,415	68,687	103,794	106,934	1087	1559	1803	1864	577,712	879,117	1,295,942	1,415,474
Sri Lanka	92	0.782	2018	4167	20,462	24,924	10	25	427	633	57,607	105,902	225,629	249,927
Sweden	3	0.945	43,172	73,369	110,770	114,515	861	1307	1440	1469	421,728	653,331	1,155,756	1,278,224
Tunisia	213	0.740	11,711	20,558	55,510	59,412	394	714	1960	2100	51,829	88,741	216,642	254,282
Turkey	74	0.820	26,047	34,966	74,809	92,124	246	351	664	811	288,986	423,309	895,106	1,109,007
United Arab Emirates	223	0.890	20,886	43,770	71,627	73,590	67	143	204	212	2,099,449	3,499,933	7,455,727	9,112,446
UK	2	0.932	36,564	62,895	99,406	128,883	1080	1849	1940	2042	806,468	1,650,892	3,950,983	4,762,083
Uruguay	9	0.817	5494	22,514	110,367	112,427	52	218	1729	1740	183,955	344,737	958,295	1,081,873
USA	1	0.926	61,604	91,537	120,368	139,161	1067	1667	1974	2272	763,725	1,165,212	1,753,221	2,069,420
Venezuela	7	0.711	3999	5256	11,828	14,164	36	52	142	170	85,033	107,288	118,517	118,568
Yemen	215	0.470	70	106	257	316	20	24	48	60	577	575	8272	8644

I, II, III and IV means four time points of data collections December 2020; March, August and October 2021, respectively

Very recently importance of socioeconomic position and Human Development Index (HDI) in study of COVID-19 has been reported [19–21]. The HDI reflects three major dimensions of human development, life expectancy at birth, education and the gross national income (PPP) per capita. Countries with higher life expectancy, income and educational levels have higher HDI values. The HDI values are calculated annually and reported by the United Nations Development Program’s Human Development Report Office. The latest report (2019) was used in the present analysis as a potential confounder.

The inbreeding coefficient means the probability that an individual has received both alleles of a pair from an identical ancestral allele and shown by *F*-value. The *F*-value, for double first cousins, first cousins, first cousin once removed, second cousins, and unrelated marriages, was 1/8, 1/16, 1/32, 1/64 and 0, respectively. The mean of *F*-values which is shown by α-value, and it is calculated as *F* = ∑PiFi, where Pi and Fi are frequency and the *F*-value of each marriage type. The α-values were estimated from data presented in the Web site www.consang.net.

Data from 65 countries were included in the study. The countries were Afghanistan, Argentina, Australia, Bahrain, Bangladesh, Belgium, Bolivia, Brazil, Canada, Chile, China, Colombia, Costa Rica, Croatia, Cuba, Czech Republic, Ecuador, Egypt, El Salvador, France, Guinea, Honduras, Hungary, India, Indonesia, Iran, Iraq, Ireland, Italy, Japan, Jordan, Kuwait, Lebanon, Libya, Malaysia, Mexico, Mongolia, Morocco, Nepal, Netherlands, Nigeria, Norway, Oman, Pakistan, Panama, Peru, Philippines, Portugal, Qatar, Saudi Arabia, Singapore, Slovakia, Slovenia, South Africa, Spain, Sri Lanka, Sweden, Tunisia, Turkey, United Arab Emirates, UK, Uruguay, USA, Venezuela, and Yemen.

Normality of variables was investigated using Kolmogrov–Smirnov test. If a variable was not distributed normally, its log-transformed or square root-transformed (SR-transformed) was used in statistical analysis. Univariable correlation analysis was used. To overcome the effects of confounders on prevalence/mortality, generalized estimating equation analysis was used. Prevalence/mortality of COVID-19 was considered as outcome variables, and the α-values as well as the potential confounders (number of performed test and HDI) were introduced into model as covariates. It should be noted that time point was used as a factor in analysis. Data were analyzed using SPSS software (version 25; SPSS Inc., Chicago, IL). Statistical analysis was performed using *P* < 0.05 as the cutoff point for significant association.

## Results

Normality test showed that HDI has normal distribution. Other variables were not distributed normally. For statistical analysis, the log-transformed of α-value, and the SR-prevalence and SR-mortality of COVID-19 and SR-number of performed test were used.

Table 2 summarizes the correlations between the study variables. It should be noted that almost all of the studies variables had significant correlation with each other. The SR-prevalence (*P* < 0.001) and SR-mortality of COVID-19 (*P*  < 0.001) were negatively associated with the log-transformed of α-value.

**Table 2:** Correlation analysis between the studied variables

Date/variables	SR-prevalence		SR-mortality		HDI	
	*r*	*P*	*r*	*P*	*r*	*P*
*End December 2020*						
Log-α value	−0.395	0.001	−0.556	< 0.001	–0.618	< 0.001
SR-performed tests	0.598	< 0.001	0.298	0.016	0.733	< 0.001
HDI	0.593	< 0.001	0.468	< 0.001	–	–
*March 19, 2021*						
Log-α value	−0.439	< 0.001	−0.603	< 0.001	–0.618	< 0.001
SR-performed tests	0.625	< 0.001	0.338	0.006	0.731	< 0.001
HDI	0.624	< 0.001	0.493	< 0.001	–	–
*August 31, 2021*						
Log-α value	−0.372	0.002	−0.498	< 0.001	–0.618	< 0.001
SR-performed tests	0.602	< 0.001	0.233	0.062	0.711	< 0.001
HDI	0.609	< 0.001	0.399	0.001	–	–
*October 25, 2021*						
Log-α value	−0.404	0.001	−0.506	< 0.001	–0.618	< 0.001
SR-performed tests	0.600	< 0.001	0.208	0.096	0.733	< 0.001
HDI	0.642	< 0.001	0.407	0.001	–	–

df is 63 for all comparisons. SR and Log mean square root- and logarithmic-transformed variables

On the other hand, there was significant negative relationship between log-transformed of α-value and HDI (*r* = −0.618, df = 63, *P* < 0.001). In all of the study time points, the SR-prevalence (P < 0.001) and SR-mortality of COVID-19 (*P* < 0.001) were significantly associated with HDI.

In order to neutralized the potential confounding effects of the HDI and number of performed tests on the correlation between α-values and epidemiologic parameters, generalized estimating equations were used (Table 3). The construction final models showed that SR-prevalence (*P* = 0.008) and SR-mortality (*P* < 0.001) parameters of COVID-19 negatively associated with the log-transformed of α-value.

**Table 3:** Results of generalized estimation equations for investigation of associations of prevalence and mortality of COVID-19 with the α-values as frequency of consanguineous marriages in the 65 countries around the world

Variables	Wald Chi-square	df	*P*-value
*SR-prevalence as dependent variable*			
Time	37.494	3	< 0.001
Log-α value	7.055	1	0.008
SR-performed tests	19.586	1	< 0.001
*SR-mortality as dependent variable*			
Time	186.856	3	< 0.001
Log-α value	17.147	1	< 0.001
Human development index (HDI)	2.466	1	0.116

## Discussion

The current study revealed that in countries with high levels of consanguineous marriages, the prevalence of COVID-19 and mortality due to COVID-19 were lower than countries having low frequency of marriage with relatives. The α-value can explain 36% of the differences observed in the mortality due to COVID-19 between different countries. Although the main finding of the current study is not consistent with previous reports which reported that primary immunodeficiency diseases [16] and risk of infection of tuberculosis and hepatitis [17] positively correlated with consanguineous marriages, it is consistent with the negative association between parental consanguinity and risk of HIV-1 infection [18].

The present finding that countries with high frequency of consanguineous marriages have low prevalence/mortality of COVID-19 might be interpreted by increased number of resistant individuals against infection SARS-CoV-2 or outcome of COVID-19 due to parental consanguinity. Consanguineous marriage results in elevation of homozygosity of mutant alleles with low frequency. Let’s assume the frequency of a given mutant allele which is resistance to COVID-19 be equal to *q* in a given population. The probability of homozygosity for this allele when marriage with biologic relatives is present and absent at population level becomes equal to *q*^2^ + *apq* and *q*^2^, respectively (*α* is the mean of inbreeding coefficient). Therefore, parental consanguinity increases the frequency of mutant homozygotes which they are resistance to COVID-19. Taken together, it is concluded that the present negative association between α-value and prevalence/mortality of COVID-19 might be a reflection of elevation of the homozygosity of several mutant alleles involved in resistance against infection of SARS-CoV-2 and/or severe form of COVID-19.

The present study is an ecologic study, and other ecologic studies have some considerations and limitations. It should be noted that frequency of consanguineous marriages and the epidemiologic parameters COVID-19 are not uniformly distributed on different parts of countries. Here average levels of consanguinity and COVID-19 epidemiologic parameters for countries were used for analysis. Hence, the present finding does not mean a causal relationship between parental consanguinity and susceptibility/mortality due to COVID-19. Several case–control and/or cohort studies are needed to confirm the present findings. Finding mutations that induced the resistance to the COVID-19 is also needed.

## Conclusions

The findings of present ecologic study revealed that countries with high frequency of consanguineous marriages, the prevalence of COVID-19 and mortality due to COVID-19 were lower than countries having low level of marriage with relatives. Considering that here the average levels of consanguinity and COVID-19 epidemiologic parameters for countries were used for analysis, the present finding does not mean a causal relationship between parental consanguinity and susceptibility/mortality due to COVID-19.

## Data Availability

All data generated for analysis are presented in Table 1 of the manuscript.

## Abbreviations

COVID-19: Coronavirus disease-2019
HDI: Human development index
SARS-CoV-2: Severe Acute Respiratory Syndrome Coronavirus 2
SR-transformed: Square root-transformed
